# Evaluation of cardiovascular risk in adult psychiatric outpatients in Qatar using two risk assessment tools

**DOI:** 10.5339/qmj.2021.27

**Published:** 2021-09-27

**Authors:** Safa Al-Rawi, Monica Zolezzi, Yassin Eltorki

**Affiliations:** ^1^Al Wakrah Hospital, Hamad Medical Corporation, Doha, Qatar E-mail: mzolezzi@qu.edu.qa; ^2^College of Pharmacy, QU Health, Qatar University, Doha, Qatar; ^3^Mental Health Hospital, Hamad Medical Corporation, Doha, Qatar

**Keywords:** cardiovascular disease, serious mental illness, risk assessment

## Abstract

Introduction: Individuals with serious mental illness (SMI) experience premature death, likely due to increased rates of obesity and cardiovascular disease (CVD). This study was conducted to estimate the CVD risk in a cohort of individuals with SMI receiving outpatient psychiatric services in Qatar and to assess contributory CVD risk factors.

Methods: This is a retrospective review of the electronic medical records of a cohort of outpatients with SMI attending a mental health clinic in Doha, Qatar. The CVD risk was estimated using two risk prediction tools: the American Heart Association and the American College of Cardiology (AHA/ACC) risk calculator and the World Health Organization/International Society of Hypertension (WHO/ISH) CVD risk prediction charts for the Eastern Mediterranean region. Descriptive and inferential statistics were used to analyze the demographic and clinical data. Data were analyzed using Statistical Package for the Social Sciences.

Results: Of the 346 eligible patients, 28% (n = 97) had obtainable data for the estimation of their CVD risk using both tools. Approximately one-third of the cohort (33%) were classified as high risk using the AHA/ACC risk calculator, and 13.3% were classified as intermediate to high risk using the WHO/ISH CVD risk prediction charts. Based on the AHA/ACC risk scores, among those with a high CVD risk, almost two-thirds had CVD modifiable risk factors (i.e., smoking, diabetes, dyslipidemia, and hypertension). No statistically significant difference in the CVD risk estimates was observed among individuals with a body mass index of more or lower than 30 kg/m^2^ (*p* = 0.815).

Conclusion: Based on the AHA/ACC risk calculator, approximately one-third of the study cohort had high CVD risk estimates. The WHO/ISH CVD risk prediction charts appeared to underestimate CVD risk, particularly for those identified as high risk using the AHA/ACC risk calculator. A closer alliance between psychiatrists and primary healthcare professionals to control modifiable cardiovascular risk factors among patients with SMI is necessary.

## Introduction

People who experience serious mental illness (SMI), particularly schizophrenia, bipolar and related disorders (BPAD) and major depression, face a heightened risk of overall mortality. Compared with the general population, the mortality rate of individuals with SMI has been reported to be two to three times higher.^1^ In addition, this increased risk is present at a younger age.^1–3^ This excess mortality can be explained not only by higher rates of accidental and nonaccidental injuries in this population, but also by a high prevalence of cardiovascular disease (CVD).^1,3–6^

The prevalence of diabetes mellitus (DM) in individuals with SMI is approximately three times higher than that in the general population.^7^ Similarly, the smoking prevalence in individuals with SMI has been reported to be fourfold higher than that in the general population.^8^ In addition, studies have reported a higher risk of hypertension, dyslipidemia, and metabolic syndrome in individuals with SMI.^9,10^ Furthermore, medications used to treat SMI cause harmful adverse cardiometabolic disturbances, including increased weight and altered glucose and lipid metabolism.^1,11,12^

There is also evidence of disparities in the provision of medical care to individuals with mental illness. The Clinical Antipsychotic Trials of Intervention Effectiveness study has shown that among approximately 1,500 patients with schizophrenia, 88% with dyslipidemia were not receiving lipid-lowering agents, 30% with DM were not receiving hypoglycemic agents, and 62% with hypertension were not receiving antihypertensive agents.^13^ A study in Qatar has reported that almost 30% of patients with SMI had at least one medical comorbidity for which inadequate medical care was provided.^14^ The results of a recent meta-analysis have demonstrated a higher CVD prevalence in individuals with SMI than that in controls, and suggested that secondary prevention of CVD was much less successful in the SMI population than that in the general population.^1^

CVD risk assessment recognizes the hazards of multiple risk factors. Absolute CVD risk is the actual risk of a specific population for developing the disease, or experiencing a cardiovascular event, within a defined period (typically 5 or 10?years).^15^ The World Health Organization (WHO) recommends CVD risk assessment as a critical step in the primary prevention of CVD for the general population.^16^ Numerous CVD risk prediction algorithms are available, mostly based on the Framingham Risk Score.^17^ They are used worldwide in CVD prevention and control efforts because they can help identify people at the highest risk of CVD events who would benefit the most from preventive interventions. Almost all CVD prevention guidelines recommend some form of risk scoring to prioritize and plan primary prevention interventions. The 2018 New Zealand Cardiovascular Disease Risk Assessment and Management for Primary Care consensus guidelines are the first to identify mental illness as an independent risk factor for CVD, and recommend CVD risk screening and assessment for individuals with SMI starting at the age of 25 years.^18^

Qatar's CVD guidelines recommend the use of the American College of Cardiology (ACC)/American Heart Association (AHA) pooled cohort risk equation to estimate CVD risk in the general population.^19^ However, published data regarding CVD risk estimates among individuals with SMI are not currently available in Qatar, making it challenging to develop effective clinical and preventive strategies. Thus, this study was conducted to estimate the CVD risk in a cohort of individuals with SMI receiving outpatient psychiatric services in Qatar and to assess the contributory CVD risk factors. In addition, this study was conducted to explore differences in CVD risk estimations between two risk scoring tools: the ACC/AHA pooled cohort risk equation, as recommended by Qatar's CVD guidelines, and the WHO CVD risk prediction charts developed explicitly for the Middle East and North Africa (MENA) regions.^20^

## Methods

This is a retrospective review of the electronic medical records of outpatients attending a mental health clinic in Doha, Qatar. The sample was a subset of a cohort of eligible research subjects of a study investigating the medical comorbidities o patients with SMI, which has been published and where the study design and subjects were described in detail.^14^ The study included individuals with schizophrenia, major depressive disorder (MDD), BPAD, and schizoaffective disorder attending an adult outpatient mental health clinic and not hospitalized at a psychiatric facility within the previous year.

Ethical approval for the main study was granted by the Institutional Review Board (IRB) of Qatar University (reference number: QU-IRB 501-E/15) and by Hamad Medical Corporation Medical Research Center (MRC) (reference number: MRC 1526/2016).

A comprehensive electronic data extraction tool using an Excel^TM^ sheet (Microsoft Corporation, Washington, United States) was used to collect patient demographic data, including gender, age, height, weight, and nationality. Laboratory data including systolic blood pressure (SBP), total cholesterol (TC), and high-density lipoprotein (HDL) cholesterol, were collected as well. Additional data including psychiatric and non-psychiatric comorbidities, current pharmacotherapy, most recent smoking status, and tobacco cessation medication were obtained. For patients with dyslipidemia on anti-lipidemic medication, a lipid profile before receiving pharmacotherapy was obtained, if documented on the medical record.

The CVD risk was estimated using the WHO/International Society of Hypertension (ISH) risk prediction charts for the MENA region, including the following variables: age, gender, SBP, smoking status, and the presence or absence of DM.^20^ Besides, for those patients with a recorded TC and HDL levels, the calculator from the AHA/ACC was also used, which included the following variables: age, gender, race, TC, HDL, SBP, whether the presence or absence of treatment for hypertension, history of diabetes, and smoking status. Height and weight were obtained to determine the body mass index (BMI). Based on the estimated BMI, the patients were divided into two categories: BMI < 30 and BMI ≥ 30 (obese).

Descriptive analyses of the demographic data and risk factors were performed. Categorical variables were expressed as frequencies and percentages, whereas continuous variables were expressed as means ±  standard deviation. The chi-square test for categorical variables and t-test for continuous variables were used to compare the demographic data and CVD risk of the cohort. Univariate analyses were conducted using Fisher's exact test to determine the characteristic factors associated with CVD risk. Only covariates with *p-*values of less than 0.05 were included in the multivariate logistic analyses to determine the independent predictors of CVD risk, based on the univariate analysis procedure described by Hosmer and Lemeshow.^21^ Results were presented as odds ratios (OR) and 95% confidence intervals (95% CI). Two-sided *p*-values of less than 0.05 were considered statistically significant for all analyses. Data analysis was performed using Statistical Package for the Social Sciences (version 23.0; IBM Corp., Armonk, NY, United States).

## Results

The medical charts of 358 outpatients were reviewed. Twelve patients (3.5%) were excluded as they had a documented history of established CVD or had a documented cardiovascular event in the past (i.e., myocardial infarct, transient ischemic attack, or stroke). Ninety-seven patients (28%) had obtainable data to estimate their CVD risk using both the AHA/ACC risk calculator and WHO/ISH CVD risk prediction charts.

As summarized in Table 1, the mean age of the patients was 41.1 ± 12.4 years (range: 15–80 years). Most patients were females (54.9%) and non-Qataris (61%). The most prevalent SMI in this cohort was MDD (50%). Approximately one-third of the cohort (35%) had at least one medical comorbidity documented (21.1% had one, and 13.9% had at least two medical comorbidities); diabetes (17.3%) was the most common medical comorbidity. Approximately two-thirds of the patients were on antipsychotics (65.3%) or antidepressants (63.9%). Table 1 displays and compares the entire cohort's characteristics and those who had obtainable data to estimate their CVD risk profile using the AHA/ACC risk calculator and WHO/ISH CVD risk prediction charts. In this table, the essential characteristics of those at a high CVD risk are highlighted.

Among the 346 outpatients with SMI included in the cohort, 281 had obtainable data to estimate their BMI. The mean BMI was 31.5 kg/m^2^ ± 6.8 (range, 14.2 − 58.0 kg/m), and 55.5% of the patients had a BMI of ≥ 30 kg/m^2^. The rate of obesity (BMI ≥ 30) was highest in patients with BPAD (72.5%), followed by those with a psychotic disorder (56%) and MDD (42.7%). No statistically significant difference in the CVD risk was found between individuals with a BMI higher and lower than 30 kg/m^2^ (*p* = 0.815).

**Figure 1.**
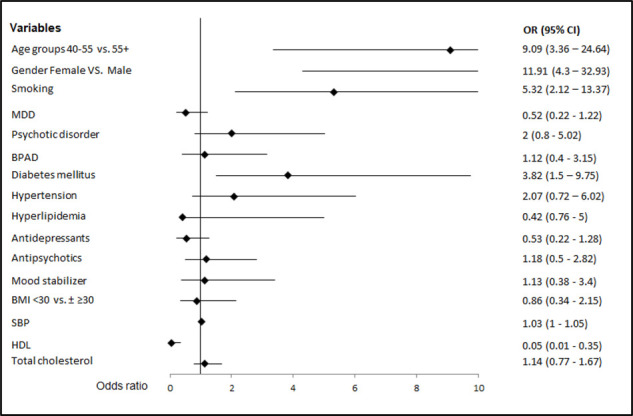
Cardiovascular risk disease risk profile of the cohort of patients with serious mental illness. The number of all SMI samples and BMI < 30 kg/m^2^ or BMI>30 kg/m^2^ with (a) high or low ASCVD risk according to the AHA/ACC risk calculator (n = 97) (b) low and moderate to high ASCVD risk using the WHO/ISH CVD risk prediction charts (n = 97)

Based on the results of the AHA/ACC risk calculator, the eligible cohort had a mean probability of developing CVD or experiencing a cardiovascular event in the next 10 years of 7.5%. Thirty-two patients (33%) were estimated to have a high atherosclerotic CVD (ASCVD) risk ( ≥ 7.5%) (illustrated in Figure 1a), of whom 59.4% (n = 19) were current smokers, 46.9% (n = 15) had DM, 45.5% (n = 10) had dyslipidemia, and 25% (n = 8) were hypertensive (see Table 1). Using the WHO/ISH CVD risk prediction charts, 11.3% of the eligible cohort (n = 11) were at a moderate to high risk ( ≥ 10%) of developing CVD, and 88.7% (n = 86) were at low risk ( < 10%) of experiencing a cardiovascular event in the next 10 years (illustrated in Figure 1b).

**Figure 2.**
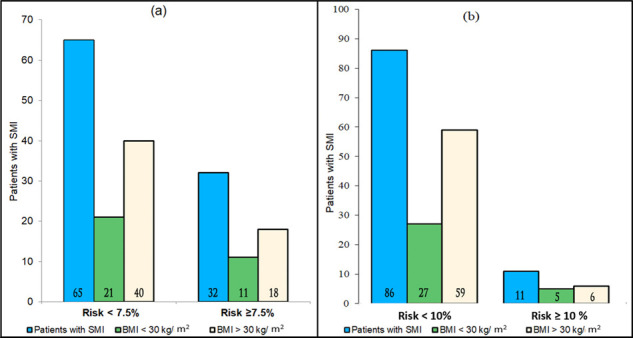
Unadjusted logistic regression analysisForest plot for univariate analysis of the characteristic risks associated with CVD risk. The bars with squares in the middle represent 95% confidence intervals (95% CIs) and odds ratios (ORs). The central vertical solid line indicates the ORs for the null hypothesis **Legend:** MDD=Major depressive disorder; BPAD=Bipolar and related disorders; BMI = body mass index; SBP = systolic blood pressure; HDL = high density lipoprotein^a^Missing data for the ASCVD risk (n = 249)^b^Missing data for smoking (n = 150)^c^Missing data for body mass index (n = 75).^*^Psychotic disorder includes schizophrenia and schizoaffective disorder

In the unadjusted logistic-regression analysis (illustrated in Figure 2), male gender (OR = 11.91; 95% CI: 4.3 − 32.93; *p* ≤ 0.0001), age of ≥ 55 years (OR = 9.09; 95% CI: 3.36 − 24.64; *p* ≤ 0.0001), DM (OR = 3.82; 95% CI: 1.5 − 9.75; *p* ≤ 0.005), receiving oral hypoglycemic treatment (OR = 3.64; 95% CI: 1.41 − 9.36; *p = *0.01), low HDL (OR = 0.05; 95% CI: 0.01 − 0.35; *p* = 0.002), receiving antilipidemic agents (OR = 2.95; 95% CI: 1.12 − 7.74; *p* = 0.03), and smoking (OR = 5.32; 95% CI: 2.12 − 13.37; *p* ≤ 0.0001) were positively associated with CVD risk.

Patients with psychotic disorders had a higher ASCVD risk than those with BPAD or MDD, with the following OR (95 % CI): 2 (0.8 − 5.02), 1.12 (0.4 − 3.15), and 0.52 (0.22 − 1.22), respectively. The results obtained from the adjusted (A) logistic regression analysis continued to show that male gender (AOR = 278; CI: 7.9 − 9844), age of ≥ 55 years (AOR = 1.5; CI: 1.2 − 1.8), low HDL (AOR = 0.001; CI: 0.0001 − 0.1), and smoking (AOR = 135.8; CI: 8.9 − 2077.1) were positively associated with CVD risk in this population.

## Discussion

This study represents Qatar's first attempt to estimate the distribution of CVD risk among a cohort of individuals with SMI attending an outpatient psychiatric clinic. Using the AHA/ACC risk assessment calculator, approximately one-third of the cohort were at a high risk of developing CVD or experiencing a major cardiovascular event in the next 10 years. These results conform to the findings of other studies in other parts of the world, which have highlighted that individuals with SMI are at a higher risk of CVD-related excess mortality.^1,6,9,22,23^ This increased risk is due in part to higher rates of CVD risk factors in this population, many of which were positively associated with a high CVD risk in this study, such as male gender, age of ≥ 55 years, DM, low HDL, and smoking. Although it did not reach statistical significance, individuals with psychotic disorders were twice more likely to have higher CVD risk estimates than those with BPAD or MDD. This finding also conforms to the results of other studies that have found that individuals with schizophrenia are at greater risk of CVD-related mortality than the general population.^1,2,5,22^

The rate of obesity in the study cohort (55.5%) is consistent with the literature, which shows numerous studies reporting that obesity is approximately twofold more common in people with schizophrenia or BPAD, and approximately 1.5 more common in individuals with depression than the general population.^4,12,24^ Interestingly, we found no statistically significant difference in the CVD risk level between individuals with higher and lower BMI, or between patients on antipsychotic medications (which are known to induce weight gain). These findings conform to other studies that have suggested that individuals with SMI have an increased CVD risk independent of metabolic disturbances, such as weight gain.^24-27^ Although some studies have suggested the possibility of a direct effect of antipsychotics on cardiovascular risk,^11-13^ we found no statistically significant difference between the use of any psychotropic agent and increased CVD risk.

Two calculators were used to estimate the CVD risk in this cohort of individuals with SMI, yielding vast differences in the CVD risk estimates. Similar results were reported in a study that has assessed the CVD risk among patients with SMI who presented after their first myocardial infarction.^27^ The CVD risk was estimated assuming that they had presented one day before the cardiac event for risk assessment. The CVD risk was calculated using six tools, including the ACC/AHA risk calculator and WHO/ISH risk prediction charts. Similar to our estimated risk scores, the WHO/ISH risk prediction charts performed worse than the ACC/AHA risk calculator in identifying those at a high risk of developing a CVD event. Although both CVD risk prediction tools are widely used and validated to estimate the CVD risk in the general population, their predictive ability in individuals with SMI has been questioned in several studies, which have reported that traditional CVD risk assessment tools tend to underestimate the CVD risk in individuals with SMI.^23,29-31^ Thus, new CVD risk prediction models specific for people with SMI are being developed and validated.^29,32^

The study's limitations correspond to its small sample size that was brought about by its retrospective nature and inadequate chart documentation of the patients’ characteristics, particularly risk factors that feed into the CVD prediction tools, making it difficult to draw generalizable conclusions. Thus, replicating this study on a larger scale is recommended, using a matched population without SMI, or using a prospective method for data collection so that comparing the observed and predicted CVD risks id possible. As these data were obtained from a cohort of patients attending a mental health outpatient clinic almost 5 years ago, further examination of cardiovascular interventions and mortality in the same cohort is needed since the initial data collection would add further light to our findings on CVD risk prediction among people with SMI. This would be a valuable comparison between the designated risk in the scales for a given group of patients and the percentage of cardiovascular interventions and mortality in this group in the last 5 years. This is a proposition for future research. Cross-sectional trials make it challenging to form a causal relationship between risk factor exposure and CVD events. A risk of bias may exist between the associations since in the cutoff points by the different calculators were observed. Until more SMI-specific and validated CVD risk assessment tools become available, it is recommended that clinicians consider multiple patient-related factors in addition to the predicted CVD risk scores when making treatment decisions.

Despite these limitations, the study highlights the importance of this work as it does lead to improved care for this vulnerable population. Recent CVD risk assessment and management guidelines are adjusting their recommendations to better inform management decisions and recommend that people with SMI have their CVD risk assessed regularly starting at the age of 25.^29^ This is an earlier age than what is recommended for the general population.

## Conclusion

To the best of our knowledge, this is the first study in Qatar that focused on CVD risk estimation in individuals with SMI. Overall, our findings conform to those from international studies on the increased susceptibility for CVD-related morbidity and mortality among patients with SMI. The lack of documentation of CVD risk factors among these patients also confirms that inequities in the provision of CVD risk assessment and management in people with SMI exist and need attention. Standard CVD risk calculators appear to underestimate CVD risk in people with SMI. Additional effort is required to integrate intrinsic determinants (as the effect of psychotropic medications or sedentary lifestyle) for CVD risk prediction in this population.

### Statement of ethics

This study complies with established guidelines for human studies according to the World Medical Association Declaration of Helsinki as evidenced by having the approval of the IRB at Qatar University (reference number: 18-060-3-016) and Hamad Medical Corporation, MRC (reference number: 16023/16).

### Disclosure statement

The authors have no conflicts of interest to declare.

### Funding sources

This study was made possible by an Undergraduate Research Experience Program (UREP) grant (UREP18-060-3-016) from the Qatar National Research Fund (QNRF) (a member of Qatar Foundation).

### Author contributions

M.Z. contributed to the conception, design, and research protocol, in addition to being involved in all aspects of data analysis and manuscript revisions for intellectual content. S.A. contributed to data collection, data analysis, and the first draft of the manuscript. Y.T. contributed to the data collection process and critically revising the manuscript. All authors approved the manuscript's submission and agreed to be accountable for all aspects of the work. The findings achieved herein are solely the responsibility of all authors.

## Figures and Tables

**Table 1:** Characteristics of adult psychiatry outpatients in Qatar using two risk assessment tools.

Characteristics	Sample (n = 346)	ASCVD risk ≥ 7.5% (n = 32)^a^	ASCVD risk < 7.5% (n = 65)^a^	WHO risk ≥ 10% (n = 11)	WHO risk < 10% (n = 86)

Age, years, mean ± SD	41.1 ± 12.4	55.1 ± 9.3	46.9 ± 6.6	60.45 ± 6.9	48.2 ± 7.7

Age groups, years, n (%)					

15–39	120 (34.7)	0 (0)	0 (0)	0 (0)	14 (16.3)

40–54	169 (48.8)	13 (18.8)	56 (81.2)	2 (18.2)	54 (62.8)

55+	57 (16.5)	19 (67.9)	9 (32.1)	9 (81.8)	18 (20.9)

Gender, n (%)					

Female	190 (54.9)	7 (12.3)	50 (87.7)	4 (36.4)	54 (62.7)

Male	156 (45.1)	25 (62.5)	15 (37.5)	7 (63.4)	32 (37.2)

Nationality, n (%)					

Qatari	135 (39)	14 (34.1)	27 (65.9)	5 (12.2)	36 (87.8)

Non-Qatari	211 (61)	18 (32.1)	38 (67.9)	6 (10.7)	50 (89.3)

Smoking, n (%)^b^	72 (36.7)	19 (57.6)	14 (42.4)	4 (36.4)	29 (33.7)

Psychiatric diagnosis, n (%)					

MDD	173 (50)	14 (26.4)	39 (73.6)	5 (9.4)	48 (90.6)

Psychotic disorder*	116 (33.5)	12 (44.4)	15 (55.6)	3 (11.1)	24 (88.9)

BPAD	70 (20.2)	7 (35)	13 (65)	3 (15)	17 (85)

Psychosis+MDD	8 (2.4)	1 (100)	0 (0)	0 (0)	1 (100)

Psychosis+BPAD	5 (1.5)	0 (0)	1 (100)	0 (0)	1 (100)

Medical comorbidities, n (%)					

Diabetes mellitus	60 (17.3)	15 (55.6)	12 (44.4)	8 (72.7)	17 (19.8)

Hypertension	45 (13)	8 (47.1)	9 (52.9)	4 (36.4)	11 (12.8)

Thyroid disease	24 (6.9)	2 (20)	8 (80)	1 (10)	9 (90)

Hyperlipidemia	43 (12.4)	10 (45.5)	12 (54.5)	2 (18.2)	17 (19.8)

CAD	6 (1.7)	1 (100)	0 (0)	0 (0)	1 (100)

Others	48 (13.9)	9 (36)	16 (64)	2 (8)	23 (92)

No. of comorbidities					

0	223 (64.5)	7 (13.2)	46 (86.8)	4 (7.5)	49 (92.5)

1	73 (21.1)	10 (45.5)	12 (54.5)	4 (18.2)	18 (81.8)

≥ 2	48 (13.9)	14 (66.7)	7 (33.3)	3 (14.3)	18 (85.7)

Psychiatric meds, n (%)					

Antidepressants	221 (63.9)	19 (28.1)	44 (71.9)	6 (9.5)	57 (90.5)

Antipsychotics	226 (65.3)	18 (29)	44 (71)	8 (12.9)	54 (87.1)

Mood stabilizers	48 (13.9)	8 (53.3)	7 (46.7)	2 (13.3)	13 (86.7)

Other psychotropics	81 (23.4)	7 (35)	13 (65)	2 (10)	18 (90)

Non-psych meds, n (%)					

Hypoglycemics	52 (15)	9 (52.9)	8 (47.1)	2 (11.8)	15 (88.2)

Anti-hypertensives	50 (14.5)	10 (62.5)	6 (37.5)	1 (6.3)	15 (93.7)

Anti-lipidemic	42 (12.1)	9 (60)	6 (40)	3 (20)	12 (80)

Thyroid agents	13 (3.8)	5 (83.3)	1 (16.7)	1 (16.7)	5 (83.3)

Other non-psychotropics	50 (14.5)	23 (57.5)	17 (42.5)	6 (15)	34 (85)

Clinical/paraclinical factors, mean ± SD					

BMI, kg/m^2^	31.5 ± 6.8	31.5 ± 6.7	31.4 ± 6.4	36.1 ± 6.7	32.5 ± 6.7

< 30 (%)	125 (44.5)	11 (34.4)	21 (65.6)	5 (15.6)	27 (84.4)

≥ 30 (%)	156 (55.5)	18 (31.0)	40 (69.0)	6 (9.2)	59 (90.8)

SBP, mmHg	127.5 ± 18.7	128.6 ± 14.4	130.1 ± 14.7	137.0 ± 18	137.0 ± 14.2

HDL, mmol/L	1.2 ± 0.4	1.2 ± 0.3	1.1 ± 0.3	1.1 ± 0.2	0.7 ± 0.3

Total cholesterol, mmol/L	5 ± 1.1	4.9 ± 0.9	5.3 ± 1.3	5.2 ± 1.3	5.3 ± 1.1


LEGEND: ASCVD = atherosclerotic cardiovascular disease; estimated CVD risk using the AHA/ACC calculator and WHO/ISH CVD risk prediction charts; MDD = major depressive disorder; BPAD = bipolar and related disorders; BMI = body mass index; CAD = coronary artery disease; SBP = systolic blood pressure; HDL = high-density lipoprotein; SD = standard deviation; meds = medications; psych = psychiatric.

a: missing data for estimating CVD risk using the AHA/ACC calculator and WHO/ISH CVD risk tables = 249 patients;

b: missing data for smoking = 150 patients; c: missing data for BMI = 7 patients (out of 97 patients) and 75 patients (out of 346 patients);

*Includes schizophrenia and schizoaffective disorder
